# The clinical features and prognosis of fungal pleural infection: A case series and literature review

**DOI:** 10.1097/MD.0000000000036411

**Published:** 2023-12-01

**Authors:** Yawan Jing, Qi Wei, Hao Zeng, Ruixin Cheng, Panwen Tian, Yalun Li

**Affiliations:** a Department of Pulmonary and Critical Care Medicine, State Key Laboratory of Respiratory Health and Multimorbidity, Precision Medicine Key Laboratory of Sichuan Province, West China Hospital, Sichuan University, Chengdu, Sichuan, China; b Lung Cancer Center, West China Hospital, Sichuan University, Chengdu, Sichuan, China; c Department of Gerontology and Geriatrics, Tibet Autonomous Region People’s Hospital, Lhasa, Tibet Autonomous Region, China.

**Keywords:** antifungal treatment, fungal pleural infection, pleural effusion, pleural fluid culture

## Abstract

Fungal pleural infections are infrequent and insidious, for which there are neither large clinical studies nor targeted guidelines to provide standardized treatment options. We reported 4 cases of fungal pleural infection and reviewed the cases of fungal pleural infections in previous studies to provide a basis for the diagnosis and treatment of fungal pleural infections. There were 2 females and 2 males with a mean age of 58.5 years in our data. The average time from onset to diagnosis was 30.25 days. Risk factors most frequently included pulmonary diseases (n = 4) and malignancy (n = 1). Two patients underwent pleural biopsy through a thoracoscope, and no pathogens were detected. Pleural fluid culture was positive in 2 out of 3 cases. The diagnoses were “possible” (n = 1), “probable” (n = 1), and “proven” (n = 2). All patients received systemic antifungal therapy, and 3 received combined thoracic drainage. The outcomes were cured (n = 1), improved (n = 2) and lost to follow-up (n = 1). We reviewed 12 cases of fungal pleural infection in previous studies. The diagnosis was confirmed via culture in 7 cases and via biopsy in 8 cases. The pathogen was *Aspergillus* in 7 cases. After a combination of systemic antifungal (n = 12) and local treatment (n = 11), 10 patients improved and 2 patients died. Diagnosis of fungal pleural infection should incorporate risk factors, clinical presentation and fungal evidence, with pleural fluid culture being an important and feasible mean of confirming the diagnosis; and treatment should be based on systemic antifungal therapy supplemented by topical therapy.

## 1. Introduction

Pleural infection is a common disease worldwide that causes serious complications, leading to increased mortality.^[1]^ Previous studies have shown a steady upward trend in its incidence.^[1–6]^ Risk factors for the increase in pleural infections may include an aging population, prevalence of chronic diseases such as diabetes, widespread application of immunosuppressive agents, an increase in severe disease and immunosuppressed status.^[7–9]^ A large retrospective clinical study showed a 3-month mortality rate of 10% and a 12-month mortality rate of 19% in patients with pleural infections.^[10]^ However, fungal etiologies of pleural infections were less common and more challenging.

Fungal pleural infection is a rare fungal infection, and its confirmation requires the detection of the fungus by histopathological or cytopathological examination, or by culture of the pleural effusion.^[11]^ Low clinical incidence and hidden site of infection are easily dismissed, and it is often combined with bacterial infections, malignancies and other immunocompromised states, leading to difficulties in diagnosis and delays in treatment.^[12,13]^ In addition, due to the low rate of positive fungal cultures of pleural effusions and the difficulty of obtaining definitive pathology, a large proportion of diagnoses are classified as “probable” or “possible”, and only a minority of patients are definitively diagnosed with a fungal pleural infection. Finally, systemic antifungal therapy may be ineffective because of the intractability of fungal pleural infections or because the patient may fail to adhere to a long antifungal regimen, leading to serious consequences. Despite this, epidemiologic and outcome data on fungal pleural infections are lacking, as fungal pleural infection is infrequent, and its microbiology can be complex.

Here, we analyzed the clinical features of 4 cases of fungal pleural infections within our hospital and reviewed the relevant literature to highlight effective diagnostic and therapeutic approaches for fungal pleurisy with the expectation of prognosis improvement.

## 2. Case presentation

We retrospectively evaluated 4 cases diagnosed with fungal pleural infection from April 2010 to April 2023 in West China Hospital. Clinical symptoms, cultivation results, admission dates, vital status, comorbidities, radiology, laboratory findings and treatment regimens were extracted. We also retrieved 12 cases of fungal pleural infections reported in previous literature between 2020 and 2023. Diagnostic criteria were based on the latest guidelines of the European Organization for Research and Treatment of Cancer and the Mycoses Study Group Education and Research Consortium (EORTC/MSGERC).^[11]^ A definitive diagnosis of fungal pleural infection requires the detection of the fungus by histopathological or cytopathological examination, or by culture of the pleural effusion.^[11]^

### 2.1. Characteristics of the 4 cases diagnosed with fungal pleural infection

**Figure 1.**
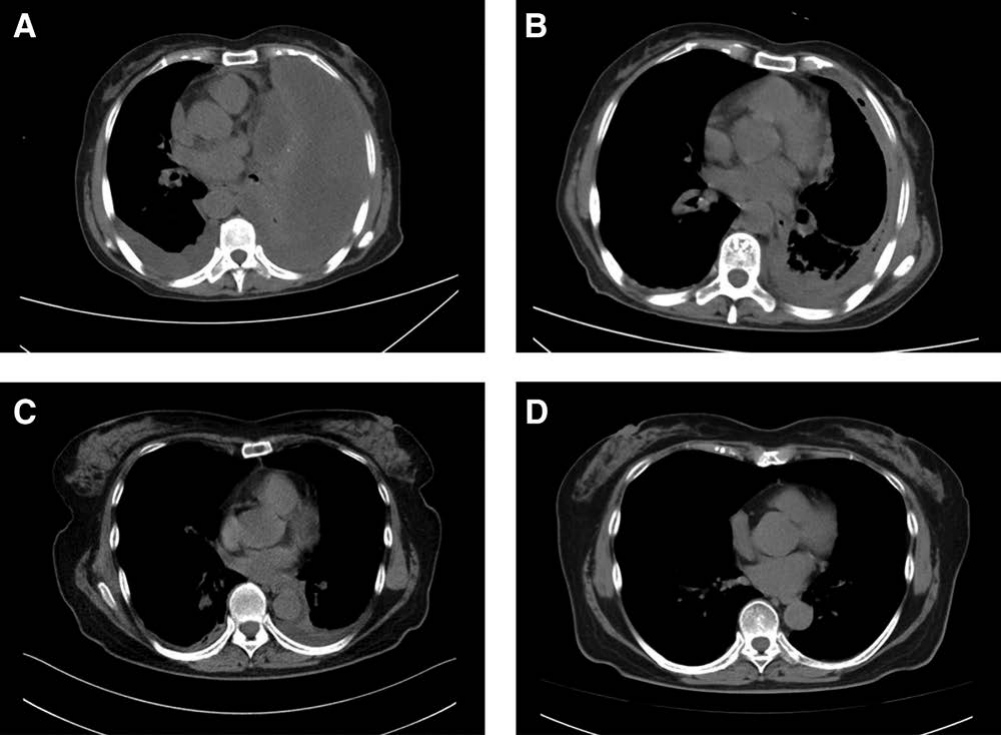
Chest CT findings in case No.1 and case No.2. Figure 1A shows the chest CT of case No.1 before treatment (January 10, 2023), and Figure 1B shows the chest CT after initiating antifungal treatment (January 26, 2023); Figure 1C shows the chest CT of case No.2 before treatment (December 17, 2022), and Figure 1D shows the chest CT after initiating antifungal treatment (January 26, 2023). CT = computed tomography.

The average age was 58.5 (27–72) years, and there were 2 females and 2 males (Table 1). All cases had combined risk factors, including 4 cases of pulmonary diseases (bronchiectasis, mycobacterial pneumonia, pulmonary infections, pulmonary schistosomiasis), 2 cases of hypoproteinemia, and 1 case of malignancy and post-chemotherapy immunosuppression. The main clinical manifestations were fever, dyspnea, chest pain, cough and sputum (Table 1). Serum G (1-3-β-D glucan) and galactomannan antigen (GM) tests were performed, and only 1 case had a positive result, while sputum cultures were conducted with positive results in 3 cases. Chest CT scan showed that case No.1 and case No.3 had pleural effusion, case No.2 was accompanied by bilateral mycobacterial pneumonia, and case No.4 had bilateral pulmonary schistosome nodules (Table 1). Figure 1 shows the results of chest CT images of case No.1 and case No.2.

**Table 1:** General clinical information of the 4 cases.

Variables	Case No.1	Case No.2	Case No.3	Case No.4
Sex	F	F	M	M
Age (yr)	66	72	69	27
Time of diagnosis	January, 2023	December, 2021	February, 2023	December, 2015
Comorbidities	Bronchiectasis, bacterial abscess thorax, hypoproteinemia, atrial fibrillation	Leukemia, post-chemotherapy myelosuppression, mycobacterial pneumonia	Pulmonary infection, hypertension	Pulmonary schistosomiasis, pulmonary mycosis, hypoproteinemia, CD4 cytopenia
Risk factors	Bronchiectasis, antibiotics	Mycobacterial pneumonia, malignancy, myelosuppression	Pulmonary infections, antibiotics, anti-tuberculosis	Parasitic infection, mycoplasma pneumonia
Clinical manifestations	Chest pain, cough, yellow pus sputum, dyspnea	Chest pain, fever	Cough, yellow pus sputum, dyspnea, chest congestion	Fever, chest pain, cough, hemoptysis
Serum G test (pg/mL)	<37 (−)	<37 (−)	<37 (−)	794.40 (+)
Serum GM test (GMI)	0.05 (−)	0.06 (−)	0.05 (−)	8.41 (+)
Sputum culture	*Candida subsmoothus, Aspergillus fumigatus complex*	Negative	*Candida subsmoothus*	*Aspergillus terreus*
Chest CT				
Location of pleural effusion	Bilateral	Bilateral	Left side	Bilateral
Pleural thickening	Yes	Yes	Yes	Yes

CRP = c-reactive protein, CT = computed tomography, F = female, G = 1-3-β-D glucan, GM = galactomannan antigen, M = male, PCT = procalcitonin, − = negative, + = positive.

### 2.2. Diagnosis, pathogenesis and pathology of the 4 cases diagnosed with fungal pleural infection

**Figure 2.**
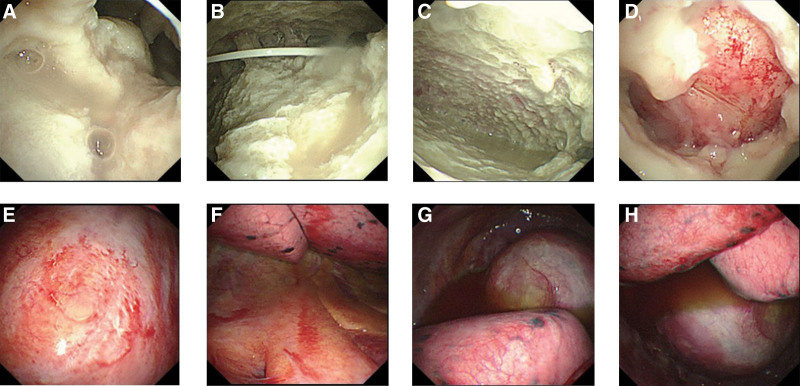
Thoracoscopic findings. Figure 2A, 2B, 2C, and 2D show the thoracoscopic findings of case No.1, which showed thickening, congestion, and erosion of the pleura in the visceral and parietal pleura with more necrotic material and adhesions on the surface, and more purulent fluid in the cavity; Figure 2E, 2F, 2G, and 2H show the thoracoscopic findings of case No.3, which showed adhesions of the pleura in the visceral and parietal pleura, with scattered sheet-like thickening and erosion of the visceral pleura, and a small amount of yellow fluid in the cavity.

According to the recommendation of the EORTC/MSGERC,^[11]^ 2 cases met the definition of proven invasive fungal disease (case No.3 and case No.4), 1 case of “probable” (case No.1) and 1 case of “possible” (case No.2) (Table 2). One of the 4 cases had an abscess in the thorax, and 2 had exudative pleural effusions. Culture of pleural effusion was positive in 2 out of 3 cases. Case No.3 was confirmed with the *Aspergillus fumigatus* complex (Table 2). Case No.1 underwent fiberoptic bronchoscopy, and the bronchoalveolar lavage fluid GM test was positive, fungal culture suggested an *Aspergillus fumigatus* complex, and cytology found filamentous fungi, with a high probability of Aspergillus. Thoracoscopy was performed in case No.1 and case No.3, both of which had pleural thickening, congestion, and erosion; case 1 had more necrotic material on the surface of the pleura and had more purulent fluid in the lumen, whereas case No.3 had only a small amount of yellow fluid in the lumen (Fig. 2). The pleura biopsy of case No.1 demonstrated proliferating fibrous tissue with acute and chronic inflammatory cell infiltration and granulation tissue proliferation in the focal area. Pleural biopsy of case No.3 demonstrated fibrous tissue hyperplasia and hyalinization with a small infiltration of chronic inflammatory cells and mesothelial cell hyperplasia in the focal area. No pathogens were found by special staining in either case (Fig. 2).

**Table 2:** The diagnosis, pathogenesis and pathology of the 4 cases.

Variables	Case No.1	Case No.2	Case No.3	Case No.4
Diagnosis method	Probable diagnosis	Possible diagnosis	Culture of pleural effusion	Culture of pleural effusion
Pleural effusion-related tests				
Appearance	Yellow and cloudy	ND	Yellow and clear	Yellow and cloudy
Number of nucleated cells (×10^6^/L)	18800	ND	70	1100
Percentage of multinucleated cells	84%	ND	90%	95%
Glucose (mmol/L)	0.01	ND	6.66	2.98
Total protein (g/L)	18.3	ND	45.2	45.6
LDH (U/L)	11653	ND	182	2658
ADA (U/L)	110.3	ND	10.4	NA
The chylus qualitative tests of pleura effusion	Positive	ND	Positive	ND
Pleural effusion culture	*Corynebacterium striatum* and *Streptococcus constellatus* subspecies	ND	*Aspergillus fumigatus complex*	Fungus
BALF	GM test 3.489GMI(Positive), Fungal culture suggesting *Aspergillus fumigatus complex*, filamentous fungi were seen, with a high probability of *Aspergillus*	ND	ND	ND
NGS of pleural effusion	Multiple bacteria can be seen; no fungus, virus or mycobacterium tuberculosis were found	ND	ND	ND

ADA = adenosine deaminase, BALF = bronchoalveolar lavage fluid, GM = galactomannan antigen, L = lymphocyte ratio, LDH = lactate dehydrogenase, N = neutrophil ratio, ND = not done, NGS = next-generation sequencing, NA = not available.

### 2.3. Treatment and prognosis of the 4 cases diagnosed with fungal pleural infection

The average time from onset to diagnosis was 37.75 (1–90) days (Table 3). The average length of residence was 26.5 (14–44) days. All cases were given systemic antifungal treatments, 3 of them received thoracentesis drainage, and 1 patient received chest irrigation with saline (Table 3). Case No.1 was treated with voriconazole (total course was 8 weeks), supplemented by intrathoracic localized treatment. Case No.2 was treated with caspofungin and posaconazole for 7 days and then maintained with posaconazole (total course was 5 months). Case No.3 was given voriconazole for 11 days and then adjusted to caspofungin for 7 days to avoid concomitant administration with rifamycins, and continued with voriconazole (total course was 8 weeks). The pleural effusion in case No.4 did not improve after 5 weeks of treatment with voriconazole. At follow-up until April 2023, the pleural effusion was absorbed in 2 cases, and the patient status was good (case No.1, case No.2); 1 case was treated with antifungal therapy for 8 weeks and still had pleural effusion, but survived well (case No.3); and 1 case was lost to follow-up (case No.4).

**Table 3:** Treatment and prognosis of the 4 cases.

Variables	Case No.1	Case No.2	Case No.3	Case No.4
Time of onset to diagnosis (days)	16	1	90	44
Time of admission to diagnosis (days)	9	4	10	14
Length of Hospitalization (days)	21	14	27	44
Systematic antifungal therapy	Total course was 8 wk: voriconazole (300 mg/12 h, IV, changed to 200 mg/12 h for 6 d after 24 h; 200 mg/12 h, oral, 7 wk)	Total course was 5 months: caspofungin (50 mg/24 h, IV, 7 d) combined with posaconazole (400 mg/12 h, oral, 9 d; 100 mg/12 h, oral, 4 + months)	Total course was 8 wk: voriconazole (300 mg/12 h, IV, 11 d) →Caspofungin (50 mg/24 h, IV, 7 d) →Voriconazole (350 mg/q12 h, oral, 5 + weeks)	Total course was 5 + weeks: voriconazole (300 mg/12 h, IV, changed to 200 mg/12 h for 13 d after 24 h; 200 mg/q12 h, oral, 21 + days)
Local treatment	Thoracoscopic surgery (saline flushing, removal of some necrotic material, insertion of a thoracic drainage tube)	None	Thoracoscopic surgery (with a thoracic drainage tube placed)	Thoracocentesis drainage
Pleural effusion status after treatment	Control	Cured	Recurring	Recurring
Outcomes	Improved	Cured	Improved	NA

NA = not available.

### 2.4. Twelve cases of fungal pleurisy were reviewed in the literature

We reviewed 12 case reports of fungal pleurisy over the previous 4 years on PubMed using the search terms “fungus and pleural effusion, fungus and pleurisy, pleural aspergillosis, fungal empyema, fungal pleurisy”, and the clinical and diagnostic characteristics were summarized in Table 4. The average age was 53.4 years (n = 11), and there were 3 females and 9 males (n = 12). All cases were combined with risk factors. The diagnosis was confirmed by culture in 7 cases and by biopsy in 8 cases. Aspergillus pleurisy was found in 7 cases. After a combination of systemic antifungal (n = 12) and local treatment (n = 11), 10 patients improved, and 2 patients died.

**Table 4:** Clinical characteristics and treatment outcomes of patients with fungal pleural infection in previous studies.

Articles	Age/Sex	Risk factors	Pathogen	Serum G-test	Serum GM-test	Systemic antifungal therapy	Surgical interventions	Outcomes
Atousa et al, 2022^[14]^	65/M	Lung cancer, pneumonectomy, chemotherapy	*Aspergillus flavus* (pleural histopathology)	NA	NA	Voriconazole	Drainage of the pleural space, open window thoracostomy procedure	Improved
Cui et al, 2022^[15]^	57/M	Diabetes mellitus, cancer, surgery	*Histoplasma* (pleural histopathology)	NA	NA	Amphotericin B → Itraconazole	VATS, chest tub, thoracic lavage with amphotericin B	Improved
Chang et al, 2022^[16]^	70/M	chronic smoker, bronchiectasis, empyema, emphysema	*Aspergillus fumigatus* (Pleural tissue culture, pleural effusion culture)	Negative	Negative	Voriconazole	Thoracentesis, VATS and partial decortication	Died
Juthipong et al, 2022^[17]^	20/M	Multiple antibiotic use in a short period of time	*Aspergillus fumigatus complex* (pleural histopathology, pleural tissue culture)	Negative	NA	Voriconazole → Isavuconazole	VATS, including drainage, decortication, pleural and rib debridement, intercostal tube placement	Improved
Zhu et al, 2021^[18]^	56/M	Diabetic, fungal empyema	*Schizophyllum commune* (pleural histopathology)	NA	NA	Voriconazole	Thoracentesis, VATS decortication	Improved
Sebahat et al, 2021^[19]^	48/M	Chronic pulmonary aspergillosis, pleural aspergilloma, bronchopleural fistulas	*Aspergillus fumigatus* (pleural histopathology)	NA	NA	Voriconazole	Endoscopic cavernostomy exploration	Improved
Joshua et al, 2021^[20]^	38/M	Hypereosinophilia, pneumonia	*Aspergillus* (pleural histopathology)	NA	Negative	Voriconazole	Evacuation of empyema, total lung decortication, and lobectomy through VATS	Improved
Hirokazu et al, 2020^[21]^	71/F	Pulmonary emphysema, interstitial pneumonia, prednisolone	*Aspergillus fumigatus* (pleural effusion culture)	Negative	Positive	Voriconazole	Chest tube and intrapleural voriconazole, fenestration surgery	Improved
Kamal et al, 2020^[22]^	NA/ M	Excessive drinking	*Candida* (pleural effusion culture)	NA	NA	Micafungin→ Fluconazole	Thoracentesis	Improved
Akshay et al, 2020^[23]^	55/F	T-cell ALL	*Cryptococcus Neoformans* (pleural effusion culture)	NA	NA	Amphotericin B →Fluconazole	Tube thoracostomy and intrapleural fibrinolysis, thoracentesis	Improved
Alan et al, 2020^[24]^	55/F	Smoker, Diabetic, inhaled marijuana	*Aspergillus niger* (pleural histopathology, pleural tissue culture)	Negative	Negative	Anidulafungin + Voriconazole → Voriconazole	Lobectomy	Improved
Chen et al, 2020^[25]^	52/M	Consumed bamboo rat meat	*Penicillium marneffei* (pleural histopathology, pleural tissue culture)	Negative	NA	Voriconazole	None	Died

ALL = acute lymphoblastic leukemia, G = 1-3-β-D glucan, GM = galactomannan antigen, NA = not available, VATS = video-assisted thoracic surgery.

## 3. Discussion

Fungal pleurisy is infrequent; however, it an important cause of death in patients with pleural infection. Previous studies showed that the overall mortality in patients with pulmonary aspergillosis increased when they developed pleural effusions^[26–28]^ and was up to 70% in fungal empyema.^[13,29]^ Nevertheless, there are few large clinical studies on fungal pleurisy. Our study summarized and analyzed the clinical features, treatment and prognosis of 16 cases (4 from in-house data, 12 from the literature) with fungal pleurisy, which is of great significance in the clinical practice of fungal pleurisy (Tables 1 and 4).

Previous studies showed that more than 47% of patients with fungal empyema had impaired immune function, 84% had combined bacterial empyema, and other high-risk factors included recent invasive chest or abdominal surgery.^[13,22,30]^ In our study, risk factors for morbidity, including pulmonary diseases, malignancy, diabetes, surgery and empyema, were present in all cases (Tables 1 and 4). Thus, fungal pleurisy tends to occur in immune-compromised patients.

Difficulty in the diagnosis of fungal pleural infections leads to delays in treatment, as reflected in prolonged hospitalization. Delayed treatment can complicate the course and increase mortality, which is a key factor in the poor outcome of pleural cavity infections.^[31]^ The G test has a limited role in the diagnosis of invasive fungal pleurisy, with only 1 positive result in 16 cases (Tables 1 and 4). Caution should be exercised when GM is found to be negative in patients treated with antifungal drugs and nonneutropenic patients.^[15]^ In our study, 9 cases (2 from in-house data, 7 from the literature) had proven mycobacterial pleurisy, while only one of them had a positive GM test (Tables 1 and 4). The EORTC/MSGERC indicated^[11]^ that confirming a diagnosis of fungal pleural infection requires fungal detection by histopathology, cytopathology, or culture of samples obtained from the chest cavity. However, the low positive culture rate of pleural effusion^[16–19]^ made it difficult to achieve an early diagnosis. One study showed that the detection rate of pleural tissue culture was much higher than that of pleural effusion or peripheral blood in patients with suspected pleural infection.^[20]^ The AUDIO study also showed that even after performing pleural tissue, pleural effusion, and blood cultures, the pathogen could not be identified in nearly 50% of patients,^[20]^ which would make the diagnosis uncertain and prevent timely selection of appropriate antimicrobial therapy, ultimately leading to delayed treatment. Of the 14 proven cases, 6 were confirmed by pleural fluid culture, 4 by pleural tissue culture, and 8 by pleural histopathology (Tables 2 and 4). In conclusion, negative results of the G test, GM test, or cultures do not completely rule out the disease. Invasive surgical biopsy should be performed when necessary to obtain a definitive diagnosis through histopathology and tissue culture.

Early initiation of systemic antifungal therapy significantly reduces the risk of death.^[13]^ Recent studies and guidelines have emphasized that treatment may be delayed while awaiting a confirmed diagnosis and that patients with an initial diagnosis of fungal infection should receive antifungal therapy as early as possible according to EORTC/MSGERC recommendations.^[15,21]^ Treatment options for different fungal infections vary. Previous studies showed that fungal pleural infection was most often caused by *Candida* followed by *Aspergillus.*^[13,22]^ In our 16 cases, 8 cases of *Aspergillus* were confirmed, 3 cases of highly suspected *Aspergillus*, and only 1 case of *Candida* (Tables 2 and 4). The drugs chosen for the first-line of treatment of Aspergillosis are isaconazole and voriconazole, and triazole antifungal drugs such as posaconazole and isoxaconazole also have good efficacy.^[23,24]^ In our study, all patients received systemic antifungal therapy (Tables 3 and 4). However, the optimal duration of maintenance therapy is not known and needs to be adjusted according to clinical manifestations, such as respiratory dysfunction and hemoptysis.^[23]^ Early initiation of systemic antifungal therapy is extremely important to improve the prognosis of patients, and appropriate empiric antifungal agents should be selected at the undiagnosed but highly suspicious phase.

The use of fibrinolytic or DNase monotherapy in adult pleural infections has been extensively studied; nevertheless, the current evidence-based basis is still insufficient.^[31]^ Previous studies have shown that patients who have failed treatment or who have accompanied treatment intolerance, refractory disease, refractory hemoptysis, poor drainage, persistent sepsis, or local complications (e.g., pleural cavity rupture) should be treated surgically after assessing the associated risks,^[25,32,33]^ such as pleurodesis, thoracoscopy, pleural debridement, thoracic lavage, and fenestration surgery lobectomy. Thoracoscopy can be selected as a screening and treatment option in complex pleural fungal infections of elderly and immunocompromised patients, but it requires a good assessment of the surgical risks as well as technical expertise, including thoracic surgery and anesthesia support.^[31]^ In our study, 7 cases were treated localized by thoracoscopic procedures (Fig. 2), such as irrigation of the chest cavity, removal of necrotic tissue from the chest cavity, and placement of chest drains, and the outcome of 6 cases were improved (Table 2, Table 4, Fig. 1). In addition, the diagnosis of 5 cases was definitively made with the pleural tissue provided by thoracoscopy, which provided a significant diagnostic and therapeutic contribution. Thoracoscopy has shown excellent effectiveness when applied to the diagnosis and treatment of pleural infections.

In terms of monitoring patient response to treatment, the European Respiratory Society and the European Society of Thoracic Surgeons (ERS/ESTS) recommended follow-up time points of 2 to 4 weeks to detect early treatment failure and 8 to 12 weeks to ensure complete radiological regression.^[31]^ However, in clinical practice, many patients are unable to achieve regular follow-up appointments due to the long antifungal course and poor efficacy of antifungal therapy in fungal chest infections. In our in-house data, 3 cases were followed up regularly, and 1 case was missed (Tables 3 and 4). Only by performing satisfactory follow-up work in the clinic can we assess the efficacy of treatment and adjust the therapeutic regimen in time to achieve a favorable prognosis.

## 4. Conclusion

Fungal pleural infections are often combined with immunocompromised status. Risk factors, clinical manifestations, and fungal evidence should be considered in clinical practice to achieve an early diagnosis, and pleural fluid culture is a feasible and important method to confirm the diagnosis. Early initiation of systemic antifungal therapy is essential to improve prognosis and may be supplemented with local therapy according to clinical practice.

## Acknowledgments

We thanked the patients in this study.

## Author contributions

**Conceptualization:** Yalun Li.

**Data curation:** Yawan Jing, Qi Wei.

**Formal analysis:** Yawan Jing, Qi Wei.

**Investigation:** Yawan Jing, Qi Wei.

**Methodology:** Yawan Jing, Qi Wei.

**Project administration:** Yawan Jing, Ruixin Cheng.

**Resources:** Yawan Jing, Hao Zeng, Ruixin Cheng.

**Software:** Hao Zeng, Ruixin Cheng.

**Supervision:** Panwen Tian, Yalun Li.

**Visualization:** Yawan Jing, Panwen Tian.

**Validation:** Hao Zeng, Panwen Tian.

**Writing – original draft:** Yawan Jing.

**Writing – review and editing:** Yawan Jing.
